# Transcriptional Analysis of *Shewanella oneidensis* MR-1 with an Electrode Compared to Fe(III)Citrate or Oxygen as Terminal Electron Acceptor

**DOI:** 10.1371/journal.pone.0030827

**Published:** 2012-02-01

**Authors:** Miriam A. Rosenbaum, Haim Y. Bar, Qasim K. Beg, Daniel Segrè, James Booth, Michael A. Cotta, Largus T. Angenent

**Affiliations:** 1 Department of Biological and Environmental Engineering, Cornell University, Ithaca, New York, United States of America; 2 Department of Biological Statistics and Computational Biology, Cornell University, Ithaca, New York, United States of America; 3 Department of Biomedical Engineering, Boston University, Boston, Massachusetts, United States of America; 4 Department of Biology, Boston University, Boston, Massachusetts, United States of America; 5 Bioinformatics Program, Boston University, Boston, Massachusetts, United States of America; 6 Bioenergy Research Unit, United States Department of Agriculture, Agricultural Research Service (ARS), National Center for Agricultural Utilization Research (NCAUR), Peoria, Illinois, United States of America; Vrije Universiteit Brussel, Belgium

## Abstract

*Shewanella oneidensis* is a target of extensive research in the fields of bioelectrochemical systems and bioremediation because of its versatile metabolic capabilities, especially with regard to respiration with extracellular electron acceptors. The physiological activity of *S. oneidensis* to respire at electrodes is of great interest, but the growth conditions in thin-layer biofilms make physiological analyses experimentally challenging. Here, we took a global approach to evaluate physiological activity with an electrode as terminal electron acceptor for the generation of electric current. We performed expression analysis with DNA microarrays to compare the overall gene expression with an electrode to that with soluble iron(III) or oxygen as the electron acceptor and applied new hierarchical model-based statistics for the differential expression analysis. We confirmed the differential expression of many genes that have previously been reported to be involved in electrode respiration, such as the entire *mtr* operon. We also formulate hypotheses on other possible gene involvements in electrode respiration, for example, a role of ScyA in inter-protein electron transfer and a regulatory role of the *cbb3*-type cytochrome *c* oxidase under anaerobic conditions. Further, we hypothesize that electrode respiration imposes a significant stress on *S. oneidensis*, resulting in higher energetic costs for electrode respiration than for soluble iron(III) respiration, which fosters a higher metabolic turnover to cover energy needs. Our hypotheses now require experimental verification, but this expression analysis provides a fundamental platform for further studies into the molecular mechanisms of *S. oneidensis* electron transfer and the physiologically special situation of growth on a poised-potential surface.

## Introduction

The *γ-*Proteobacterium *Shewanella oneidensis* MR-1 attracts broad attention because of its unusual respiratory versatility. Besides respiration with oxygen, *S. oneidensis* is capable of anaerobic respiration with many different terminal electron acceptors, such as nitrate [1], dimethyl sulfoxide (DMSO) [2], iron(III) [3], [4], electrodes [5], [6], [7], [8], and uranium(VI) [9] and other toxic heavy metals [10], [11], [12], [13], [14]. The respiratory capabilities with heavy metal compounds make *S. oneidensis* a very attractive microbe for bioremediation applications [3], [15], since the reduced metals are often immobilized and less toxic than the oxidized forms. In addition, respiration with an electrode as electron acceptor has gained great interest in the emerging bioengineering discipline of bioelectrochemical systems (BESs) [6], [7], [16]. BESs can exploit the ability of *S. oneidensis* to transfer metabolic electrons from organic substrates to an electrode [17], [18].

For bioremediation and BESs, a thorough understanding of the biochemical reaction mechanisms is required to optimize the microbe's electron transfer rates. Many biochemical and genetic studies have been performed to clarify the reaction mechanisms of extracellular respiration [7], [19], [20], [21]. It is clear that for most reaction steps of metal reductions, *c*-type cytochromes play key roles and different electron acceptors require different sets of cytochromes. While single or multiple deletion mutants verified some of these cytochromes for specific reaction steps (e.g., CymA or MtrC), the presence of 42 possible *c*-type cytochromes in the *S. oneidensis* genome makes conclusive knock-out mutation experiments difficult, because protein functions may be substituted with alternative cytochromes (e.g., MtrA and its paralogues MtrD and DmsE, [21]). Today, the general respiratory pathway in *S. oneidensis* is known as follows: lactate, which is the primary substrate, is oxidized to acetate, carbon dioxide, and four electrons. These electrons are stored in a menaquinone pool within the cytoplasmic membrane of the cell from where they are passed on to a versatile inner membrane *c*-type cytochrome CymA – a reductase. This in turn interacts with a broad range of *c*-type cytochromes involved in many of the above mentioned respiratory pathways [2], [12], [22], [23], [24], [25]. For solid, external electron acceptors (e.g., metal minerals, electrodes) and for soluble iron(III) citrate [21], a chain of cytochromes has to transfer the electrons across the outer membrane. In this case, the periplasmic reductase MtrA passes the electrons on to reductases that are associated with the outer membrane (through MtrB [a noncytochrome] to MtrC and OmcA, respectively), which in turn perform the terminal reduction of the extracellular electron acceptor. Although the direct involvement of these enzymes in soluble or insoluble iron(III) and electrode respiration has been demonstrated [7], [21], [26], [27], other enzymes may substitute their function if the respective genes are deleted [21], resulting in a complex interaction network of reductases during respiration of *S. oneidensis*.

Transcriptional profiling with genomic microarrays to study the gene expression levels could provide a more global insight in the physiological activity of *S. oneidensis* during extracellular respiration. Beliaev et al. performed two studies regarding gene expression during anaerobic respiration with various electron acceptors [28], [29]. In the first study, mRNA levels from aerobically grown cells were compared to anaerobic respiration with fumarate, soluble iron(III), and nitrate [28]. The predicted *c*-type cytochromes CymA, MtrA, MtrB, and OmcA were upregulated with all three anaerobic electron acceptors compared to oxygen respiration; and the highest expression levels were detected during fumarate respiration. Because of the highest expression with fumarate, the gene expression with various metal and nonmetal electron acceptors was analyzed relative to fumarate respiration under anaerobic conditions in a second study [29]. The expression of the *mtrCAB* operon was increased 2- to 8-fold with fumarate compared to all metal-reducing conditions (including nonsoluble forms). To the best of our knowledge, *S. oneidensis* gene expression with an electrode as electron acceptor has not yet been evaluated. This might be due to the experimental challenges of performing transcription analyses of *S. oneidensis* electrode biofilms, which grow very slowly under completely anaerobic, continuous conditions (barely a monolayer of cells after 2–3 weeks of biofilm growth, Figure S1).

Transcriptional studies with *Geobacter sulfurreducens*, which is also intensively studied for its extracellular electron transfer capabilities, gave important insight into possible protein involvement in electrode respiration [30], [31]. Holmes et al. [30] confirmed some genes that had been predicted for extracellular respiration, while other predicted genes showed no increased expression with an electrode compared to soluble iron(III) as the electron acceptor. However, the *Geobacter* biofilm is typically fairly thick (>50 µm) and different physiological states at different distances from the electrode surface have been identified [32], which makes true transcriptional profiling very difficult to interpret without experimental finesse. In contrast, the thin monolayer biofilms of *S. oneidensis* on carbon paper electrodes under anaerobic conditions (Figure S1 and [33]) should not be prone to physiological effects of substrate or pH gradients, as they are encountered in thick *G. sulfurreducens* biofilms [32], [34].

Thus, here we took a global approach to evaluate physiological activity of *S. oneidensis* and performed gene expression analysis with Affymetrix Gene Chips to compare, for the first time, the overall gene expression with an electrode to soluble iron(III) as the electron acceptor (Comparison 1) and an electrode to oxygen as the electron acceptor (Comparison 2). The expression analysis is complemented with experimental performance data, such as optical density, iron(III) reduction activity, and current generation. We focused our transcription analysis on: i) genes involved in energy metabolism; ii) biofilm physiology; and iii) stress response activity. Since we compared the physiological state of very different growth conditions, the statistical analysis was challenging. Therefore, we employed a new statistical package for our microarray analysis that is especially powerful at small sample sizes [35]. It is important to note that lacking the detection of differential expression for a certain gene does not necessarily mean that this gene was not differentially expressed, but rather that a clear, statistically-sound conclusion was not possible. In addition, we are aware that transcriptional data may not represent the true physiological state of the microorganism, since post-transcriptional processing (translation and functional protein regulation) is not reflected by this analysis. Therefore, we use this microarray study as a tool to discover new gene expression – phenotype relationships, which serve as a platform for further experiments to verify physiological activities.

## Results

### Performance of *S. oneidensis* with an electrode, iron(III) citrate, or oxygen as electron acceptor

We operated two bioelectrochemical reactors to study *S. oneidensis* electrode respiration with lactate as the electron donor for 2 weeks. Steady-state performance was reached after ∼6 days with an average current density of 26±5 µA/cm^2^ and coulombic efficiency of 20±4% (averaged over 8 days and both trials). Under anaerobic operation (the electrode as the only available electron acceptor) at an average hydraulic retention time of 6.5 h, we observed very little planktonic growth (OD_600 nm_<0.1). In addition, scanning electron microscopy images showed only a thin monolayer of *S. oneidensis* cells on the carbon paper electrode (Figure S1). The liquid cultures with iron(III) citrate and oxygen as electron acceptors reached an optical density (OD_600 nm_) of 0.54±0.03 and 0.80±0.01, respectively, after 20 h of growth at 30°C. By the end of the experiment, the iron(III) citrate culture consumed 45% of the available substrate and 80% of the available electron acceptor (41 of 50 mM iron[III] citrate) with a coulombic efficiency of 72% (ratio of electrons consumed to electrons used to reduce iron[III] to iron[II]). Thus, cultures respiring with an electrode and soluble iron(III) were harvested at a late-logarithmic time point (steady-state conditions in a continuous bioreactor corresponds to late-logarithmic growth). On the other hand, the aerobic culture consumed most of the substrate (<1% lactate remained) and was in the early stationary growth phase when the cells were harvested.

### Gene expression analysis of *S. oneidensis* MR1

**Figure 1:**
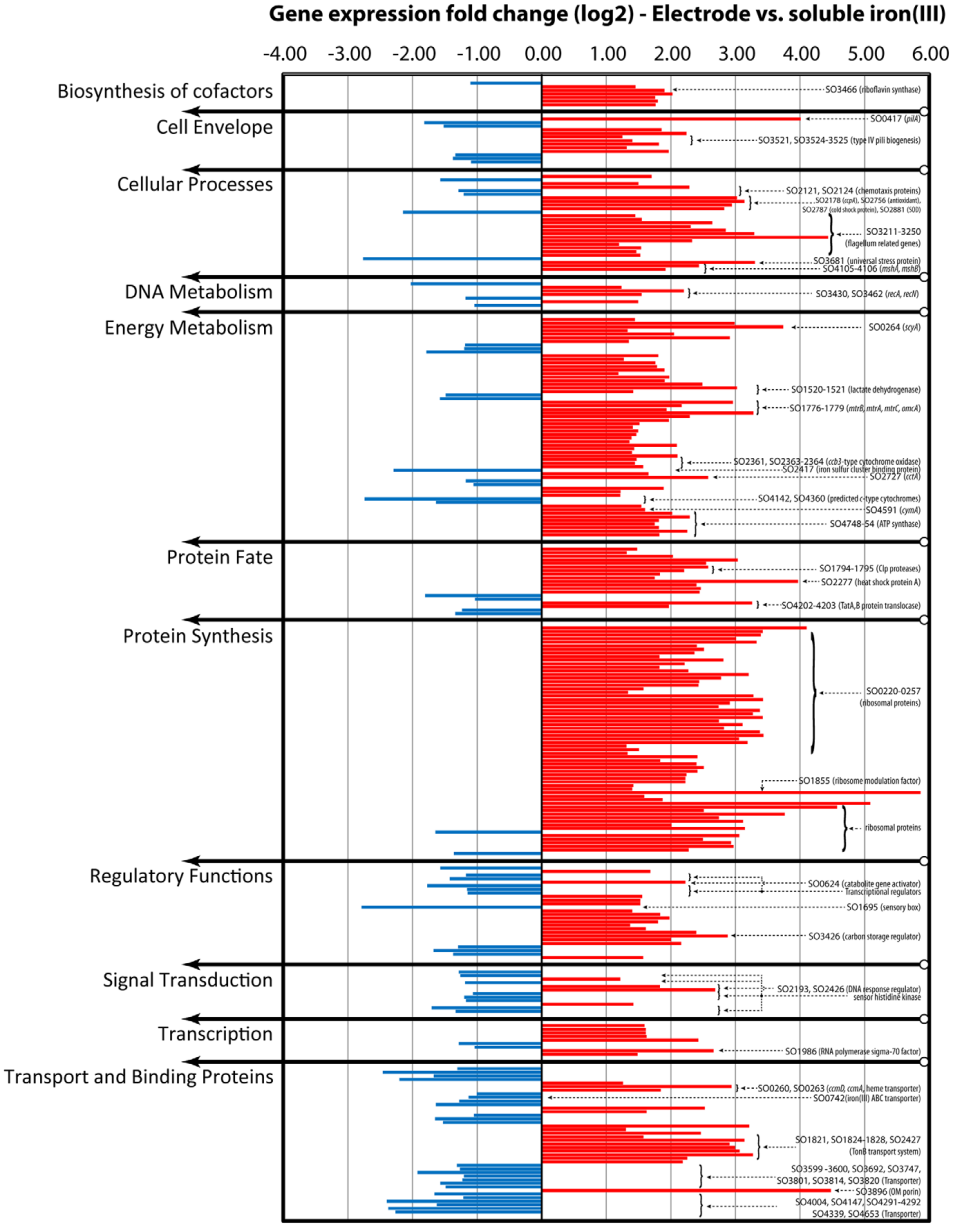
Differential expression levels of selected genes in comparision 1 (El vs. Fe). Bar diagram showing the over and under expression of selected genes with the electrode in Comparison 1 (electrode vs. soluble iron[III]). The selection includes transcripts of interest with q≤0.05 and |logFC|<1, which have been designated by * in Table S1. Genes are clustered into functional groups. Most genes discussed in the text have been labelled.

**Figure 2:**
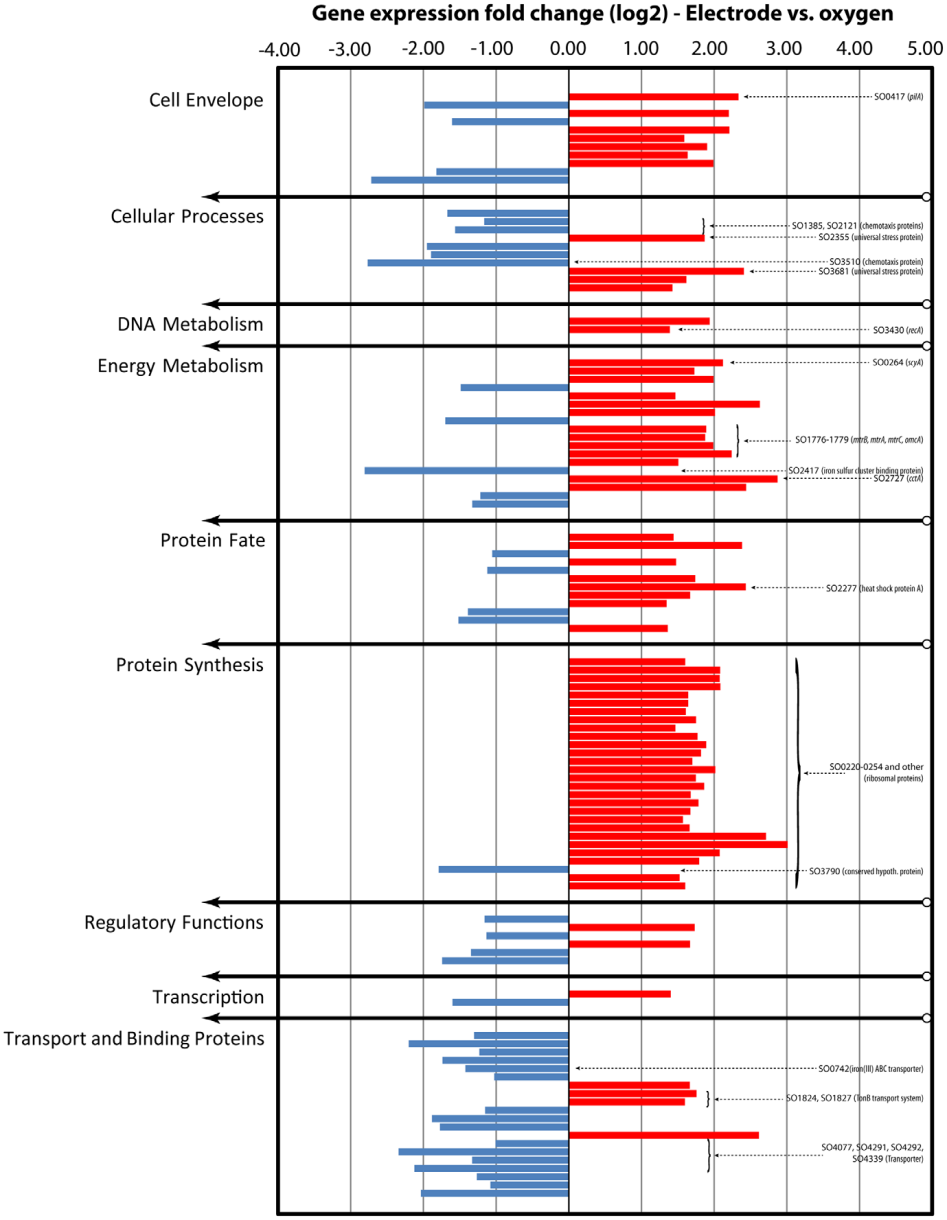
Differential expression levels of selected genes in comparision 2 (El vs. O2). Bar diagram showing the over and under expression of selected genes with the electrode in Comparison 2 (electrode vs. oxygen). The selection includes transcripts of interest with q≤0.2 and |logFC|<1, which have been designated by * in Table S2. Genes are clustered into functional groups. Most genes discussed in the text have been labelled.

We performed gene transcript analysis for 3949 *S. oneidensis* genes with Affymetrix gene chips in two statistical comparisons to determine the differential gene expression: **Comparison 1** relates the transcript with the electrode (“El”, at 0.4 V vs. standard hydrogen electrode [SHE] working electrode potential; number of biological replicates [gene chips] n = 2) to iron(III) citrate (“Fe”, n = 4) as the electron acceptor; and **Comparison 2** relates the transcript with the electrode (at 0.4 V vs. SHE) to oxygen (“O_2_”, n = 3) as the electron acceptor. A summary of the comparison results is given in Table 1 for false discovery rates (fdr) of q≤0.05 and q≤0.2. Graphical illustrations of the overall differential gene expression in both comparisons is given in Figure S2 and a graphical illustration of the statistical model fitting, which was used for analysis, is given in Figure S3. Selected differentially expressed genes that are relevant to our discussion are clustered into functional groups and presented in Figures 1 and 2.

**Table 1:** Summary of gene expression analysis of a total of 3949 genes of *S. oneidensis*.

Comparison	Sample size (n_test_;n_control_)	Variance mean(m_g_)^2^	Number of detected genes	
			fdr≤0.05	fdr≤0.2
El vs. Fe	2;4^1^	0.15	919	1357
El vs. O_2_	2;3	0.28	42	193

1 2 of the 4 Fe samples were grown without the 5 g/L sodium ß-gycerolphosphate, which was present in all other media; statistical tests confirmed no difference in expression within those samples.

2 The mean(m_g_) represents the average (across all genes) mean-squared error for each performed comparison.

### Comparison 1: Respiration with a carbon electrode vs. soluble iron(III) (El vs. Fe)

When we compared the gene expression of *S. oneidensis* with the electrode poised at 0.4 V vs. SHE to iron(III) citrate as electron acceptor, we found a total of 918 differentially expressed genes (at q≤0.05) of which 674 had a |logFC|>1 (Table S1, selected genes in Figure 1, logFC = log[2] of the expression fold change). Seventy percent of these 674 genes were upregulated during respiration with an electrode. 13 down- and 59 up-regulated genes were involved in energy metabolism (Table 2). Of the downregulated genes, three genes represented unspecified *c*-type cytochromes, which were all located in the genome adjacent to other genes encoding cytochrome *c* proteins. Among the upregulated genes were 11 predicted *c*-type cytochromes, 12 dehydrogenases, and 7 of the 9 subunits of the ATP synthase (SO4748–4754, SO4746 [epsilon subunit] did not show differential expression [q = 0.83], SO4747 [beta subunit] differential expression was not statistically significant [q = 0.15]) (Figure 1 – “Energy Metabolism”). All genes for proteins of the proposed electron transfer chain during extracellular respiration of *S. oneidensis* – CymA, MtrA, MtrB, MtrC, and OmcA (all but MtrB are *c*-type cytochromes) [36] – were upregulated with logFC>1 during extracellular respiration with the electrode (Table 2), even though some of these proteins are also known to be involved in soluble iron(III) reduction (CymA [23], MtrA [37], and MtrC [21]). In addition, we detected *cctA* (cytochrome *c3*) to be upregulated, which previously has been related to extracellular respiration activity in a *mtrA*-knockout strain [21]. Further, we detected higher transcript levels of the monoheme cytochrome *c* ScyA (SO0264), which is an unspecified diheme cytochrome *c* (SO4485), and the three subunits of a *cbb3*-type cytochrome *c* oxidase during electrode respiration. We also found higher expression levels of genes coding for proteins involved in cytochrome *c* biosynthesis (SO0259, SO0260, SO0263 – *ccmE*, *ccmD*, and *ccmA*, respectively [38]).

**Table 2:** Differently expressed genes related to “Energy Metabolism” in Comparison 1 (electrode [@ 0.4 V vs. SHE] vs. Fe(III) as electron acceptor) with |logFC|>1.

logFC	Gene ID	Gene description	Adjusted p-value	Comments
1.445606	SO0049	phosphoglycerate mutase	0.0302	Glycolysis/Gluconeogenesis
2.986795	SO0259	cytochrome *c* biogenesis protein CcmE	0.0000	Cytochrome *c* biosynthesis
3.736397	SO0264	ScyA	0.0000	ScyA, monoheme cytochrome *c5*
1.329064	SO0292	ribulose-phosphate 3-epimerase	0.0329	
2.049366	SO0401	alcohol dehydrogenase	0.0001	
2.91455	SO0406	thioredoxin 1	0.0000	
1.351289	SO0432	aconitate hydratase 2	0.0307	
−1.187912	SO0476	CcmG-2	0.0009	
−1.197626	SO0478	CcmF-2	0.0088	
−1.783812	SO0716	hypothetical monoheme cytochrome *c*	0.0000	
1.807039	SO0770	malate dehydrogenase	0.0000	TCA-cycle
1.501327	SO0780	glycine cleavage system H protein	0.0007	
1.381005	SO0930	Transketolase	0.0083	
1.270539	SO0932	phosphoglycerate kinase	0.0308	Glycolysis/Gluconeogenesis
1.760373	SO0970	FccA	0.0058	Complex II, furmarate reductase
1.782208	SO1013	NADH dehydrogenase I	0.0008	Complex I, NADH dehydrogenase
1.898404	SO1020	NADH dehydrogenase I	0.0104	Complex I, NADH dehydrogenase
1.186554	SO1105	NqrC-2 NADH:ubiquinone oxidoreductase	0.0419	
1.974139	SO1200	triosephosphate isomerase	0.0000	Glycolysis/Gluconeogenesis
1.904597	SO1429	anaerobic dimethyl sulfoxide reductase	0.0423	
2.487641	SO1490	alcohol dehydrogenase II	0.0001	Ethanol metabolism
−1.4943	SO1495	putative glycosyl hydrolase	0.0005	
3.021139	SO1520	Fe-S oxidoreductase, L-lactate dehydrogenase	0.0296	Lactate metabolism
1.413225	SO1521	D-lactate dehydrogenase (dld)	0.0035	Lactate metabolism
−1.487265	SO1538	isocitrate dehydrogenase	0.0000	TCA-cycle
1.61607	SO1678	methylmalonate-semialdehyde dehydrogenase	0.0132	
−1.572969	SO1748	hypothetical protein	0.0075	One type c heme binding motif
2.960933	SO1776	MtrB	0.0000	Outer membrane protein MtrB
2.167019	SO1777	MtrA	0.0001	Outer membrane cyt. *c*, MtrA
1.930627	SO1778	MtrC/OmcB	0.0000	Outer membrane cyt. *c*, OmcB
3.277322	SO1779	OmcA	0.0000	Outer membrane cyt. *c*, OmcA
2.290059	SO1926	citrate synthase	0.0000	TCA-cycle
1.969943	SO1928	succinate dehydrogenase	0.0001	TCA-cycle, Complex II
1.51245	SO1929	succinate dehydrogenase	0.0351	TCA-cycle, Complex II
1.41192	SO1932	succinyl-CoA synthase	0.0115	
1.495658	SO1933	succinyl-CoA synthase	0.0010	
1.89302	SO1962	MelA	0.0077	
1.466551	SO2222	fumarate hydratase	0.0053	TCA-cycle
2.163865	SO2304	alanine dehydrogenase	0.0002	
1.388457	SO2330	flavodoxin	0.0083	
1.355206	SO2336	phosphoglucomutase	0.0066	Glycolysis/Gluconeogenesis
2.091597	SO2339	alpha keto acid dehydrogenase complex	0.0000	
1.43161	SO2340	alpha keto acid dehydrogenase complex	0.0147	
1.398345	SO2341	alpha keto acid dehydrogenase complex	0.0129	
2.103882	SO2345	glyceraldehyde 3-phosphate dehydrogenase	0.0000	Glycolysis/Gluconeogenesis
1.469091	SO2361	CcoP	0.0014	*cbb3*-type cytochrome *c* oxidase
1.444194	SO2363	CcoO	0.0133	*cbb3*-type cytochrome *c* oxidase
1.571258	SO2364	cytochrome c oxidase, CcoN	0.0020	*cbb3*-type cytochrome *c* oxidase
−2.29432	SO2417	iron-sulfur cluster-binding protein	0.0303	
1.316717	SO2487	6-phosphogluconate dehydratase	0.0147	
1.328647	SO2489	glucose-6-phosphate 1-dehydrogenase	0.0230	
1.653075	SO2491	pyruvate kinase II	0.0057	Glycolysis/Gluconeogenesis
1.594663	SO2638	leucine dehydrogenase	0.0127	
2.578122	SO2727	STC small tetraheme cytochrome *c*	0.0000	Periplasmic cytochrome *c3* (CctA)
−1.175027	SO2912	PflB	0.0297	Pyruvate-formate-lyase
−1.055207	SO3034	ferric iron reductase protein	0.0158	
−1.359768	SO3057	histidase family protein	0.0488	
1.88887	SO3285	cytochrome d ubiquinol oxidase	0.0020	
1.220109	SO3420	monoheme cytochrome *c*	0.0474	
1.214519	SO3517	NADH dehydrogenase	0.0217	Complex I, NADH dehydrogenase
−1.178324	SO3662	FixG-related protein	0.0000	
−2.741183	SO4142	hypothetical monoheme cytochrome *c*	0.0058	
−1.634153	SO4360	MtrAD-like decaheme cytochrome *c*	0.0001	
1.542187	SO4485	diheme cytochrome *c*	0.0045	unknown cyt. *c*, membrane anchored
1.600531	SO4591	CymA	0.0011	periplasmic cytochrome *c*, CymA
2.018634	SO4748	ATP synthase F1	0.0000	Complex V, ATP synthase
2.291916	SO4749	ATP synthase F1	0.0000	Complex V, ATP synthase
1.813468	SO4750	ATP synthase F1	0.0010	Complex V, ATP synthase
1.746106	SO4751	ATP synthase F0	0.0043	Complex V, ATP synthase
1.812044	SO4752	ATP synthase F0	0.0023	Complex V, ATP synthase
2.25591	SO4753	ATP synthase F0	0.0000	Complex V, ATP synthase
1.821587	SO4754	ATP synthase protein I	0.0055	Complex V, ATP synthase

A complete - searchable and resortable - table of all differentially expressed genes in this comparison is given in Table S1.

Since electrode-respiring cells were grown in a biofilm, but iron(III) citrate-respiring cells were in planktonic culture, we found pili genes, which are required for cell attachment on the electrode, at higher expression levels than for the planktonic culture (*pilA*: SO0417, logFC = 4.0074; type IV pili biogenesis genes: SO3521–3528, logFC: 1.2782–1.9647; MshA and MshB pilin: SO4105–4106, logFC = 2.4267 and 1.9187; Figure 1 – “Cell Envelope”). We also detected 11 flagellum related genes (within flagellum locus SO3211 to SO3255) to be upregulated in the biofilm at the electrode (Figure 1 – “Cellular Processes”).

Two recently identified lactate dehydrogenases (SO1520 and SO1521) [39] also showed higher expression with the electrode than with soluble iron(III) as electron acceptor, as did many of the core enzymes of the TCA-cycle (see Table 2), indicating a higher metabolic activity in cells grown with the electrode as electron acceptor. The detected dehydrogenases include members of the microbial respiration chain: succinate dehydrogenase (SO1929) and NADH dehydrogenase I – subunits B+J (SO1020 and SO1013), and two alcohol dehydrogenases (SO0401 and SO1490).

Besides genes involved in energy metabolism, many genes related to protein synthesis, protein degradation, and stress response were found to be upregulated in the biofilm at the electrode (Figure 1 – “Protein Synthesis”, “Protein Fate”, and “Cellular Processes”). Many highly upregulated genes with the electrode coded for ribosomal proteins (SO0220 to SO0257). Corresponding to this high expression level of protein synthesis genes in the electrode biofilm, we also found transcripts for many protein degrading proteases and peptidases highly upregulated compared to iron(III) citrate respiration (e.g., Clp proteases [SO1794, logFC = 2.5759; SO1795, logFC = 2.2022; SO2626, logFC = 2.4645; SO3577, logFC = 2.4440; for more examples on peptidases see Table S1]). Further, various genes involved in temperature or oxidative stress response showed higher expression in the *S. oneidensis* biofilm at the electrode. Some examples are SO2756 – designated as an antioxidant (logFC = 3.1374), SO2787 – cold shock protein (logFC = 2.9453), SO2881 – superoxide dismutase (logFC = 2.8270), SO3681 – universal stress protein (logFC = 3.2994), SO2016 – heat shock protein (logFC = 1.8294), and SO2277 – heat shock protein (logFC = 3.9683) (Figure 1 – “Cellular Processes”).

Other genes with very high |logFC| values in Comparison 1 (see Table S1) code for the major outer membrane lipoprotein (SO1295, logFC = 6.3943), an outer membrane porin (SO3896, logFC = 4.4747), a ferrous iron transport protein A (SO1783, logFC = 3.2121), and the Sec-independent protein translocases TatA and TatB (SO4202–4203, logFC = 3.2583 and 1.9701). Several TonB system transport proteins (and adjacently located transport protein genes; SO1821, SO1823–1829) and two, thus far, unspecified TonB-dependent receptor proteins (SO2427, SO2907) showed elevated mRNA levels during electrode respiration.

### Comparison 2: Respiration with a carbon electrode vs. oxygen (El vs. O_2_)

When comparing the gene expression of an electrode biofilm grown at 0.4 V vs. SHE with oxygen as the electron acceptor, we found that 42 genes were differently expressed at q≤0.05. To increase our sensitivity with the aim to increase the number of detected genes, we performed additional statistical testing to evaluate the statistical quality of predictions at a lower false discovery rate level. We found 188 genes at q≤0.2 (Table S2 with |logFC|>1; selected genes are shown in Figure 2). Despite the increase of the expected false positive rate to 20% (i.e., out of 100 predicted nondifferentially expressed genes in our comparison, we now detect 20 instead of 5 genes as differentially expressed), our analysis showed that the predicted true positive rate at the same time is increased to ∼40% (i.e., we now detect 40 out of 100 predicted truly differently expressed genes) (explanation in Figure S3Bc). Thus, the probability of detecting a truly differently expressed gene is double as high as for detecting a false positive gene and our chances to discover new gene expression – phenotype relationships are increased.

For the q≤0.2 settings, we found 19 differently expressed genes that are involved in energy metabolism (Table 3, Figure 2 – “Energy Metabolism”). Thirteen genes had higher transcript levels with the electrode and six genes with oxygen as terminal electron acceptor. Proteins known to be involved in extracellular respiration (CctA, MtrA, MtrB, MtrC, and OmcA) were found to be upregulated with the electrode as electron acceptor, as were a decaheme cytochrome *c*, which is designated as a DMSO reductase (SO1427, DmsE), a fumarate reductase (SO0970), and the unspecified monocytochrome ScyA (SO0264). Further, the pyruvate-formate-lyase (PflB, SO2912) displayed increased expression with the electrode as electron acceptor. On the other hand, we observed an iron-sulfur cluster binding protein (SO2417), a putative monoheme cytochrome *c* (SO0716), and an unspecified (MtrAD-like) decaheme cytochrome *c* protein (SO4360) to be upregulated with oxygen as electron acceptor. The aforementioned ribosomal gene cluster (Comparison 1), several peptidases, and stress proteins showed increased transcription levels with the electrode compared to oxygen as electron acceptor (Table S2, Figure 2 – “Protein Synthesis”, “Protein Fate”, and “Cellular Processes”).

**Table 3:** Differently expressed genes related to “Energy Metabolism” in Comparison 2 (electrode [@ 0.4 V vs. SHE] vs. oxygen as electron acceptor) with |logFC|>1.

logFC	Gene ID	Gene description	Adjusted p-value	Comments
2.1214	SO0264	ScyA	0.0236	ScyA, monoheme
1.7277	SO0401	alcohol dehydrogenase	0.1422	Fermentation
1.9917	SO0406	thioredoxin 1	0.0871	
−1.4853	SO0716	hypothetical monoheme cytochrome *c*	0.0714	
1.4668	SO0770	malate dehydrogenase	0.1115	TCA-cycle
2.6280	SO0970	FccA	0.0519	TCA-cycle, Fumarate reductase
2.0109	SO1427	MtrAD-like decaheme cytochrome *c*	0.1115	
−1.6389	SO1495	putative glycosyl hydrolase	0.0981	
−1.6984	SO1748	hypothetical protein, one heme binding motif	0.1115	
1.8899	SO1776	MtrB	0.0871	Outer membrane protein
1.8762	SO1777	MtrA	0.1711	Cytochrome *c*
1.9874	SO1778	MtrC/OmcB	0.1422	Outer membrane cytochrome *c*
2.2403	SO1779	OmcA	0.1115	Outer membrane cytochrome *c*
1.5080	SO2339	alpha keto acid dehydrogenase complex	0.1422	
−2.8072	SO2417	iron-sulfur cluster-binding protein	0.1135	
2.8713	SO2727	STC small tetraheme cytochrome *c*	0.0380	Periplasimic CctA
2.4403	SO2912	PflB	0.0252	Pyruvate-formate-lyase
−1.2157	SO3034	ferric iron reductase protein	0.1935	
−1.3272	SO4360	MtrAD-like decaheme cytochrome *c*	0.1364	

A complete (searchable and resortable) table of all differentially expressed genes in this comparison is given in Table S2.

## Discussion

### Broad physiological differences between the growth conditions affect statistical testing

Performing satisfactory mRNA extraction of thin *S. oneidensis* electrode biofilms is a difficult task and comparing the physiological activity of *S. oneidensis* electrode biofilms to common liquid cultures with soluble electron acceptor includes several major physiological changes: i) biofilm formation and maintenance vs. suspended liquid culture growth; ii) continuously-fed chemostatic growth vs. shaken batch culture; and iii) the difference in terminal electron acceptor: electrode, soluble iron(III) citrate, or oxygen. Yet, while standard Affymetrix microarray analysis software was not suitable to analyse such difficult datasets due to insufficient sensitivity at small sample sizes, new statistical tools based on hierarchical models [35], [40] provide sufficient analytical power to deliver meaningful results. Expectedly, broad changes of the overall gene expression patterns were found in our expression analysis (graphical illustration in Figure S2), which makes the statistical identification of specific differently expressed genes challenging. Because of this broad bandwidth of changes in the genome for our comparisons and our relatively small sample sizes, the gene-specific variance of our results (estimated by the gene-specific mean squared error, which we denote m_g_; Figure S3), and therefore the resolution of our analysis varied. The average (across all genes) mean-squared error, mean(m_g_), of Comparison 1 is only half as high as for Comparison 2 (mean[m_g_] = 0.15 vs. 0.28). Consequently, Comparisons 1 and 2 had a very different statistical resolution and showed very different significant expression changes. Thus, 918 vs. 42 genes were statistically detected to have changed in expression level for Comparison 1 (El vs. Fe) and Comparison 2 (El vs. O_2_), respectively (if controlling the false discovery rate for both at 5%), although we would expect broader expression changes for Comparison 2. Yet, by using advanced statistical methods, we found many genes to be differentially expressed with the electrode compared to soluble iron(III) or oxygen, despite the experimental limitations that challenged the statistical analysis. Microarray expression analyses are just a screenshot of the physiological state of an organism, and thus to evaluate the true physiological state, our hypotheses now require experimental verification. We see this study as a road map for further physiological investigations and extensive follow-up research based on the herein detected differential expression and our resulting hypotheses are currently under way in our lab.

### Extracellular respiration with cytochrome *c* protein machinery

For Comparisons 1 (El vs. Fe) and 2 (El vs. O_2_), we detected the cytochrome *c* protein machinery, which was predicted to be responsible for extracellular respiration, to be more highly expressed with the biofilm electrode [36]. In Comparison 1, we detected transcripts for all proteins known to be involved in this electron transfer chain: CymA, MtrA, MtrB, MtrC/OmcB, and OmcA, and in Comparison 2, we found the genes for MtrA, MtrB, MtrC/OmcB, and OmcA to be more highly expressed with the electrode as electron acceptor. We also detected the gene of the periplasmic reductase CctA to be upregulated during electrode respiration, although it has been found that CctA only seems to have a physiological role in combination with MtrD (which was not detected) in the *absence* of MtrA [21]. Interestingly, we found significantly elevated expression of four reductases (*cymA*, *mtrA*, *mtrB*, and *mtrC*) during electrode respiration although the gene products are also required for soluble iron(III) citrate reduction [21]. Many of our results (e.g., the enhanced expression of genes of central metabolism, protein synthesis, or stress responds, Figure 1) indicate that the overall metabolic activity with the electrode was higher than with soluble iron(III), while the growth was lower (see discussion below), which could explain the significantly increased expression of these reductase genes with the electrode vs. iron(III) citrate. We did not detect elevated expression levels of *cymA* when we compared electrode respiration vs. oxygen respiration. However, this could be a result of the lower resolution of the statistical analysis for this comparison. The mean log2 expression values for *cymA* are 11.3 with the electrode, 10.0 with soluble iron(III), and 10.1 with oxygen. Thus, the expression values with the electrode are ∼2.5 times higher than expression with either soluble iron(III) or oxygen, but statistically this gene is only recognized in Comparison 1 (El vs. Fe).

### Possible role of other *c*-type cytochromes

In addition to established cytochromes, other *c*-type cytochromes (SO0264, SO0716, SO3420, and SO4485) were detected to be differentially expressed with the electrode compared to soluble iron(III) and/or oxygen as electron acceptor. In both comparisons, we found the gene *sycA* (SO0264) that encodes for a small, soluble periplasmic monoheme cytochrome *c* to be differentially expressed (El vs. Fe: logFC = 2.59, El vs. O_2_: logFC = 1.47). Its location in the genome (within the *ccm* cytochrome *c* maturation gene cluster SO0259–269) and a transcription study by Beliaev et al. [29] suggest its involvement in cytochrome *c* maturation. The gene has also been designated as a cytochrome *c5* and it shows high homology to cytochrome *c5* proteins from other microorganisms, including *Idiomarina* and *Vibrio* species [41]. It was reported to have increased expression with fumarate compared to nitrate respiration [1] and to be a very abundant cytochrome especially under oxygen limited conditions [41]. Meyer et al. [41] suggested that the cytochrome *c5* (SycA) might function as an electron mediator between cytochrome complexes, and it might function as electron donor to the bacterial cytochrome *c* peroxidase (BCCP [CcpA], SO2178), which also showed enhanced expression in our Comparison 1 (El vs. Fe; logFC = 3.020, p = 0.048).

A hypothetical *c*-type cytochrome (SO0716), a (noncytochrome) ferric iron reductase (SO3034), and a MtrAD-like decaheme cytochrome (SO4360) were more highly expressed with soluble iron(III) and oxygen than with the electrode, indicating that they may not be involved in electrode respiration. Bretschger et al. [7] found increased activity for electrode, manganese(IV) oxide, and insoluble iron(III) reduction with a SO0716 mutant. They also tested a SO4360 mutant and found lower activity for electrode respiration, but slightly higher activity for manganese(IV) oxide and insoluble iron(III) reduction (compared to the *S. oneidensis* wild type activity).

With the electrode, we also detected elevated expression levels for MtrAD-like decaheme cytochrome *c* DmsE (SO1427, detected in Comparison 2) and MtrB-like DmsF (SO1429, detected in Comparison 1), which belong to the designated DMSO reductase system. Gao et al. [42] and Coursolle et al. [21] documented a dominating role of DmsE in DMSO respiration with mutant studies. However, upregulation of the DMSO reductase genes in *S. oneidensis* with electron acceptors other than DMSO has been reported (thiosulfate, fumarate, soluble iron(III), and nitrate) [28], [29]. MtrA/MtrD mutant experiments revealed a minor role of DmsE in replacing their functions for soluble and solid iron(III) respiration [21]. Deletion of *dmsE* did not or only slightly impact growth with other natural electron acceptors of *S. oneidensis* [42], [43], but instead led to an increase of current production, solid iron(III), manganese oxide, and DMSO reduction compared to the wild type strain [7], [43], which was explained through an increased expression of the outer membrane DMSO reductases DsmA and DsmB in the Δ*dmsE* mutant [43]. Further investigations of DsmE and DsmF are required to clarify their exact function in extracellular respiration processes.

This again shows the complexity of the *S. oneidensis c*-type cytochrome network, especially with reference to different electron acceptors. While most catabolic reactions of glycolysis and TCA are substrate and product specific, the energy conserving reactions of *c*-type cytochromes are redox-potential driven and the formal potential of the electron donor and acceptor under the respective conditions might be more crucial for the reaction than the chemical identity of the reacting species.

### Microbial cell attachment in the biofilm

Several genes involved in cell attachment to form a biofilm showed elevated expression levels in Comparison 1 (Figure 1 – “Cell Envelope”, “Cellular Processes”). Thormann et al. [44] reported that *S. oneidensis* initial monolayer biofilm formation is mediated by MSHA pili, which we found upregulated in our electrode experiments (SO4105 and SO4106). Later, the same group showed that the *mxdABCD* genes are required besides the MSHA pili for multilayered, three-dimensional biofilm formation [45]. We did not detect an upregulation of the *mxdABCD* genes, which is in agreement with the monolayer biofilm of *S. oneidensis* observed in our experiments (Figure S1). We found upregulation of *pilA* (SO0417) during electrode respiration (logFC = 4.0074 compared to soluble iron[III], logFC = 2.3341 compared to oxygen). Although PilA is required for attachment and extracellular respiration in *G. sulfurreducens*, it was not yet shown to be essentially involved in these processes in *S. oneidensis*. A recent study on *S. oneidensis* biofilm formation showed that deletion of *pilA* did not have a phenotypic effect on MSHA pili-mediated cell attachment [46]. Although *pilD* has been shown to be crucial for electrode respiration, likely because of its involvement in type II secretion [7], [47], it was not detected to be differentially expressed in our tests.

Although our expression analysis was performed with mRNA isolated from the electrode *biofilms*, we detected strong expression of many flagellum-related genes in Comparison 1 (11 out of 39 flagellum related genes in locus SO3211–SO3255). Thormann et al. [44] found that many motility genes (flagellum-related genes) are required for early biofilm formation, and transposon mutants deprived of these gene functions were not able to form a biofilm. Other work suggests that *Shewanella*'s extracellular respiration is related to flagellum controlled motility in a process called electrokinesis in which *Shewanella* cells can approach the extracellular electron acceptor in a “touch-and-go” pattern [6]. The thin and open biofilm structure observed in our experiments (Figure S1), supports both discussed functions of flagella in *Shewanella* sp.: i) ongoing initial attachment for biofilm formation; and ii) possible electrokinetic behaviour.

### Increased metabolic turnover and stress response with the electrode as terminal electron acceptor

Our gene expression results indicate that the metabolic activity of the cells with the electrode as electron acceptor might be higher than with iron(III) citrate or oxygen. Indeed, in both comparisons we saw statistically significant differences in the expression of the ribosomal protein gene cluster. The order of expression levels was: El (electrode respiration)>O_2_ (oxygen respiration)>Fe (soluble iron respiration) with an average logFC (relative to Fe) of 2.77 ∶ 1.23 ∶ 0 for genes SO0220 to SO0257, indicating the highest ribosomal activity for the electrode biofilm, followed by oxygen respiration and then soluble iron(III) respiration. Besides the ribosomal gene cluster, many genes involved in central energy metabolism, protein synthesis and degradation, or stress response showed highest expression with the electrode. Thus, while the strongest growth (OD_600 nm_) was found for oxygen respiration, we found indications for the highest metabolic activity for electrode respiration, where a very sparse layer of microbes at the electrode surface and a very low planktonic optical density of the continuous-flow media indicated very low growth rates (after 2 weeks of growth under continuous conditions). A low cell density because of cells being washed-out in the continuous electrochemical experiments is not likely, because we also found an OD_600 nm_<0.1 in the planktonic media of 50-h batch electrochemical experiments (unpublished results from a different study under the same media and electrode conditions).

While slow stationary phase-like growth rates and increased stress tolerance are commonly observed in microbial biofilms, the high metabolic turnover in our electrode biofilm experiments was puzzling. One possible explanation for a much higher metabolic turnover with the electrode and the activity of strong stress response mechanisms (inclusive protein regeneration and DNA repair mechanisms) would be the presence of a significant stress factor. This could limit efficient energy conservation during electrode respiration, and therefore enhance the metabolic turnover to sustain cell functions. Microbial respiratory reactions are driven by the redox-potential difference between electron donor and acceptor. The electrode was poised at 0.4 V vs. SHE, while the approximate redox potential for the reduction of soluble iron(III) to iron(II) at pH 6.5 is 0.1–0.2 V vs. SHE (derived from Eh – pH diagrams [48]). Thus, theoretically the energy gain for the bacteria should be higher with the electrode than with soluble iron(III). However, the coulombic efficiency – as a measure of how many electrons of a substrate undergo respiration (i.e., energy conservation) with the electron acceptor – was 3.6 times higher for iron(III) citrate than for electrode respiration (72% vs. 20%, respectively). Several studies concerning the *S. oneidensis* stress response have pointed out that despite (or because) of its metabolic versatility, *S. oneidensis* shows much higher stress sensitivity than other organisms [29], [49]. With the variety of upregulated stress response genes (e.g., oxidative stress, heat stress, transporter genes), the specific type of stress factor on *S. oneidensis* growth and physiology in our experimental setup is not known at this point. Research to identify and quantify this metabolic stress factor is currently underway in our laboratory; for example, we will quantify the metabolic and stress response activity of electrode biofilms grown at different applied electrode potentials.

### What is the role of the *cbb3*-type cytochrome *c* oxidase?

In Comparison 1, we detected an upregulation of the *cbb3*-type cytochrome *c* oxidase genes with the electrode as electron acceptor (SO2361–2364). In general, *cbb3*-type cytochrome *c* oxidases are considered to be involved in oxygen reduction reactions, however anaerobic functions have also been identified, especially in *Rhodobacter* sp. [50], [51]. In *Rhodobacter*, the oxidase is expressed under microaerobic and anaerobic conditions with a high affinity for oxygen to regulate (repress) the expression of photosynthetic genes in the presence of oxygen. A reductant flow through the *cbb3*-system under anaerobic conditions was also suggested for *Rhodobacter sphaeroides* [50]. A transcriptional study with a *S. oneidensis* mutant of the electron transport regulator EtrA, which was performed under anaerobic conditions, showed elevated expression levels of the *cbb3*-system when the regulator was present [52]. Thus, our expression results for the *cbb3*-type cytochrome *c* oxidase under anaerobic conditions (N_2_/CO_2_ over pressure) fit well in a line of other anaerobic studies concerning *S. oneidensis* or *R. sphaeroides*. However, the nature of its function under anaerobic conditions (e.g., oxygen scavenging, regulation of gene expression, or response to oxidative stress) remains to be investigated. *S. oneidensis* deletion mutants of the *cbb3*-type oxidase genes behaved similar to the wild type towards electrode, solid manganese, and iron oxide reduction [7], but showed compromised growth with soluble chromium(VI) as electron acceptor [42].

**Concluding**, this work does not try to prove new enzymatic or regulator functions related to differently expressed genes in *S. oneidensis*. It rather develops hypotheses for potential gene expression – phenotype relationships as a roadmap for further research. Our biggest findings in this microarray study are indications to a significant stress situation of *S. oneidensis* under electrode respiration conditions. The strong electropositive field that is typically applied in BES applications might challenge *Shewanella*'s natural stress responds and result in enhanced metabolic turnover and limited biofilm formation capability. Current research in our lab is following up on this hypothesis. Besides this, we were able to correlate the expression of various individual genes (e.g., *sycA*, *cctA*, *dsmE*, or the *cbb3* oxidase genes) to previous research findings and formulate new hypothesis for their function.

## Methods

### Strains and media for bioelectrochemical systems

*S. oneidensis* MR-1 (a gift from Tim Gardner, Boston University, Boston, MA, USA) was grown in LB medium for strain maintenance; culture stocks were stored with 40% glycerol at −80°C. The medium for all experiments was prepared according to Myers and Nealson [53] and was modified by adding 1.27 mM K_2_HPO_4_, 0.73 mM KH_2_PO_4_, 125 mM NaCl, 5 mM HEPES, 0.5 g/L yeast extract, 0.5 g/L tryptone, and 5 g/L sodium ß-glycerophosphate (no addition of amino acids as in the original medium recipe). The pH was adjusted to 7.2. After autoclaving, sodium L-lactate was added to final concentrations of 20 mM. In iron respiration experiments 50 mM iron(III) citrate (Sigma-Aldrich, St. Louis) served as the electron acceptor. Analytical chemicals were ACS grade.

### BES reactor operation

Two identical H-type electrochemical reactors were made of glass with an anode and cathode liquid chamber volume of 220 mL each. The anode and cathode chambers were separated by an anion exchange membrane (19.6 cm^2^, AMI-7001 Membranes International, Glen Rock, NJ, USA). Each anode chamber (working electrode chamber) was temperature controlled at 30°C with a water jacket, stirred, continuously fed with minimal medium at an hydraulic retention time (HRT) of 5–10 h, and was equipped with a carbon paper electrode (4×6.25 cm, AvCarb P50, The Fuel Cell Store, San Diego, CA, USA). It was bound to a graphite rod (Poco Graphite, Inc., Decatur, TX, USA) with carbon cement (CCC Carbon Adhesive, EMS, Hatfield, PA, USA). The cathode chambers (counter electrode chamber) were operated in batch mode with a graphite block electrode (3×9×1 cm, PocoGraphite, Decatur, TX, USA). We used an Ag/AgCl (saturated KCl) reference electrode to control the anode potential at 0.2 V vs. Ag/AgCl with a potentiostat (VSP, Biologic, Knoxville, TN). The entire assembled setup, including two 10-L feeding tanks, was autoclaved before the experiment. The tanks were used consecutively, so that each tank fed both anode chambers simultaneously. At all times, the medium tanks and the reactors were kept anaerobic by applying a positively pressured 20% CO_2_/80% N_2_ atmosphere. After background measurement for one day, *S. oneidensis* biofilms were grown to steady state. At electrode sampling time, 5×5 mm pieces of the electrode were fixed, prepared for, and imaged via SEM imaging according to [33]. Biofilm RNA was collected for transcriptional analysis.

### Chemical Analysis

Filtered samples (0.2-µm nitrocellulose filter, Millipore, Billerica, MA, USA) were analyzed for organic acids using a SpectraSYSTEM liquid chromatography system equipped with a refractive index detector (Thermo Fisher Scientific, Pittsburgh, PA) and with an organic acids column (Aminex HPX-87H Column, Bio-Rad Laboratories, Inc., Hercules, CA, USA). Samples were run at 65°C and eluted at 0.6 mL/min with 5 mM sulfuric acid. Optical density of the planktonic cultures was measured in triplicate at 600 nm with a 96-well plate reader (Synergy4, BioTek Instruments, Inc., Winooski, VT, USA). For determining iron(III) reduction, iron(II) was quantified with a Ferrozine-assay modified after Ruebush et al. [54]. To assay, 100 µL of sample were mixed with 33 µL of 2 N hydrochloric acid. After centrifugation (5 min @ 10,000 g), 50 µL of the acidified sample were combined with 950 µL Ferrozine dye (1 g/L Ferrozine in 100 mM HEPES, pH 7), mixed, and absorbance was measured in triplicate at 562 nm with a plate reader (as above) and compared to a freshly prepared iron(II) standard curve that was processed like the samples.

### Microarray analysis

#### Chemicals and reagents used for microarrays

All general chemicals for molecular biological work have been described in [55].

#### RNA sampling and isolation

RNA for microarray analysis was sampled from two bioelectrochemical systems and seven planktonic cultures (4 with iron(III) citrate and 3 with oxygen). On day 14 of the operation, the carbon paper electrodes were removed from the bioelectrochemical system, bathed in Qiagen RNA-protect for 30 s, and immediately frozen at −80°C. The biofilm samples were loosened (scraped) from the carbon electrode with a sterile razor blade. Then, the electrode was washed with 2 mL RNA protect and the biofilm-carbon sludge was transferred to a 15-mL tube. 7 mL of ice-cold phosphate buffered saline (PBS) was added; the mix was vortexed on highest speed and centrifuged for 10 min at 5,500 g. The supernatant was replaced with 7 mL ice-cold PBS, sonicated at 7 W for 30 s on ice, and centrifuged (three repetitions). Planktonic cultures were grown for 20 h. 2 mL of each culture were combined with 2 mL RNA protect, vortexed, and centrifuged at 5,500×g for 10 min. Then, the pellets of all samples (biofilm and planktonic) were resuspended in 0.75 mL NAES buffer (50 mM sodium acetate buffer, 10 mM EDTA and 1% SDS at pH 5). RNA was isolated with a phenol∶chloroform extraction protocol as reported by Cury and Koo [56]. The isolated RNA was purified from genomic DNA contaminations with Ambion DNase I treatment following the manufacturer's instructions. RNA yields were quantified with a NanoDrop spectrometer (Thermo Scientific, Pittsburgh, PA) and UV 260/280 ratios were calculated to check purity of each RNA sample. RNA quality was verified in a 1.5% agarose electrophoresis gel with ethidium bromide staining.

#### Microarray hybridization

A previously described protocol [57] was used for microarrays on *S. oneidensis* chips from Affymetrix Inc.. In short, approximately 10 µg of each RNA sample was used for cDNA synthesis, cDNA purification, and cDNA-fragmentation. This was followed by labelling of cDNA and 16 h of hybridization at 45°C on *S. oneidensis* arrays. The labelled arrays were subjected to several cycles of washing and staining using Affymetrix Wash buffers A and B, Goat IgG, Streptavidin, Anti-streptavidin and SAPE according to the Affymetrix protocol for prokaryotic arrays. This was followed by scanning of the stained arrays with Affymetrix GeneChip Scanner Model 3000.

#### Statistical analysis

The microarray data were pre-analyzed with Microarray Suite version 5.0 (MAS 5.0) using Affymetrix default analysis settings and global scaling as normalization method. Since we worked with a very small sample size of n = 2–4 for our environmental conditions, we applied the new R-based statistical package LEMMA (Laplace approximated EM Microarray Analysis) to the normalized data for the analysis of our microarray data [35] (http://www.stat.cornell.edu/lemma/, available from http://cran.r-project.org/ by Bar and Schifano, 2009). The advantage of LEMMA, which is a hierarchical model [40], over other microarray analysis tools is its high power in simulations, combined with a low false discovery rate for differentially expressed genes. We accounted for the large number of hypotheses (i.e., genes tested; 3949 of 4077 spotted genes on the chip, after removal of misreads) by using the Benjamini-Hochberg adjustment to the p-values, which allows control of the false discovery rate (fdr) at any desired level [58]. We declared a gene to be significantly differentially expressed if its adjusted p-value was smaller than a certain threshold, and the absolute value of the logarithm (base 2) fold change (logFC) was greater than 1. Data visualization was performed using the statistical software JMP8.0.

#### Microarray data publication

The *Shewanella oneidensis* MR-1 platform and microarray data has been submitted to and published with the Gene Expression Omnibus (NCBI, series accession number: GSE20379).

## Supporting Information

Figure S1 **Electron microscopic image of a monolayer** ***S. oneidensis*** **electrode biofilm.** SEM image (at 2000×) of anaerobic *S. oneidensis* on a carbon paper anode grown at 0.4 V vs. SHE.
(PDF)

Figure S2 **Illustration of genome-wide gene expression fold change.** Panel A: Results of Comparison 1 – El vs. Fe; Panel B: Results of Comparison 2 – El. vs. O_2_. With **grey**: Genes that did not change expression levels with statistical significance (fdr (q)-value threshold ≤0.05 in (A) and ≤0.2 in (B)) or where |logFC|<1. Colored dots show significantly changed genes of the following functional groups: **red** - “Energy Metabolism”; **green** - “Protein Synthesis”; **blue** – “Protein Fate”; **black** – all other functional groups. Positive log_2_ Fold Change (logFC) values indicate increased expression with the electrode, negative logFC values indicate increased expression with the other electron acceptor.
(PDF)

Figure S3 **Statistical analysis of Affymetrix Gene Chips with the LEMMA hierarchical model.** Summary of statistical analysis for comparisons 1 (Panel A) and 2 (Panel B): a) Expression effect represented as a Log2 Fold change (dg); b) The distribution of gene-specific mean squared error; c) Predicted ratio of false positive to true positive rate for the respective comparison. The false positive rate is defined as the number of detected unchanged (“null”) genes in relation to the total number of null genes at the chosen statistical cut-off (adjusted p-value). The true positive rate is defined as the number of detected differentially-expressed genes (“non-null”) in relation to the total number of non-null genes at the chosen statistical cut-off.
(PDF)

Table S1 **Differentially expressed genes with fdr≤0.05 and |logFC|>1 for Comparison 1: respiration with a carbon electrode (0.4 V vs. SHE) vs. soluble iron(III) citrate; sorted by the Gene-ID.**
(XLS)

Table S2 **Differentially expressed genes with fdr≤0.2 and |logFC|>1 for Comparison 2: respiration with a carbon electrode (0.4 V vs. SHE) vs. oxygen; sorted by the Gene-ID.**
(XLS)
