# Supplementary material for: Transcriptional Analysis of Shewanella oneidensis MR-1 with an Electrode Compared to Fe(III)Citrate or Oxygen as Terminal Electron Acceptor
Source: PLoS One. 2012 Feb 1;7(2):e30827. doi: 10.1371/journal.pone.0030827 (PMC3271074; doi:10.1371/journal.pone.0030827)
Supplement: Figure S1 — Electron microscopic image of a monolayer S. oneidensis electrode biofilm. SEM image (at 2000×) of anaerobic S. oneidensis on a carbon paper anode grown at 0.4 V vs. SHE. (PDF) [file pone.0030827.s001.pdf]

Figure S1. Electron microscopic image of a monolayer *S. oneidensis* electrode biofilm.

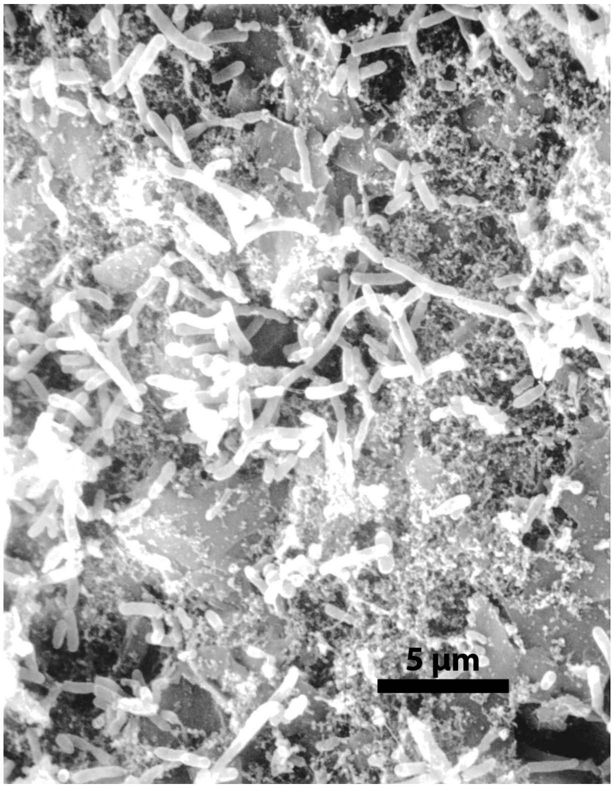

SEM image of anaerobic *S. oneidensis* on a carbon paper anode grown at 0.4 V vs. SHE.
