# Supplementary material for: Transcriptional Analysis of Shewanella oneidensis MR-1 with an Electrode Compared to Fe(III)Citrate or Oxygen as Terminal Electron Acceptor
Source: PLoS One. 2012 Feb 1;7(2):e30827. doi: 10.1371/journal.pone.0030827 (PMC3271074; doi:10.1371/journal.pone.0030827)
Supplement: Figure S2 — Illustration of genome-wide gene expression fold change. Panel A: Results of Comparison 1 – El vs. Fe; Panel B: Results of Comparison 2 – El. vs. O2. With grey: Genes that did not change expression levels with statistical significance (fdr (q)-value threshold ≤0.05 in (A) and ≤0.2 in (B)) or where |logFC|<1. Colored dots show significantly changed genes of the following functional groups: red - “Energy Metabolism”; green - “Protein Synthesis”; blue – “Protein Fate”; black – all other functional groups. Positive log2 Fold Change (logFC) values indicate increased expression with the electrode, negative logFC values indicate increased expression with the other electron acceptor. (PDF) [file pone.0030827.s002.pdf]

Figure S2. Illustration of genome-wide gene expression fold change.

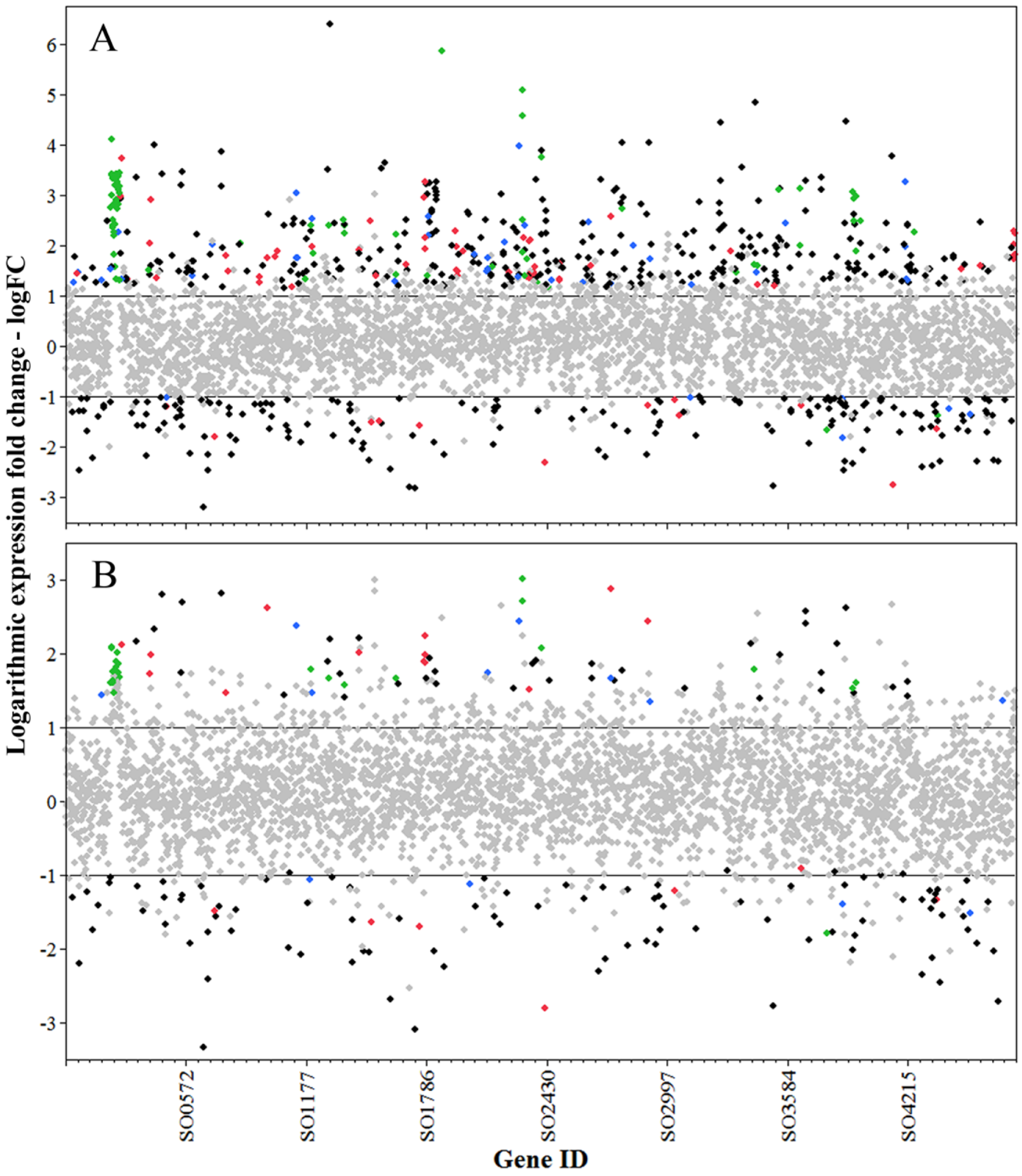

Panel A: Results of Comparison 1 – El vs. Fe; Panel B: Results of Comparison 2 – El. vs. O_2. With grey: Genes that did not change expression levels with statistical significance (fdr (q)-value threshold ≤ 0.05 in (A) and ≤ 0.2 in (B)) or where |logFC| < 1. Colored dots show significantly changed genes of the following functional groups: **red** - "Energy Metabolism"; **green** - "Protein Synthesis"; **blue** – "Protein Fate"; **black** – all other functional groups. Positive $\log_2$ Fold Change (logFC) values indicate increased expression with the electrode, negative logFC values indicate increased expression with the other electron acceptor.
