# Supplementary material for: Transcriptional Analysis of Shewanella oneidensis MR-1 with an Electrode Compared to Fe(III)Citrate or Oxygen as Terminal Electron Acceptor
Source: PLoS One. 2012 Feb 1;7(2):e30827. doi: 10.1371/journal.pone.0030827 (PMC3271074; doi:10.1371/journal.pone.0030827)
Supplement: Figure S3 — Statistical analysis of Affymetrix Gene Chips with the LEMMA hierarchical model. Summary of statistical analysis for comparisons 1 (Panel A) and 2 (Panel B): a) Expression effect represented as a Log2 Fold change (dg); b) The distribution of gene-specific mean squared error; c) Predicted ratio of false positive to true positive rate for the respective comparison. The false positive rate is defined as the number of detected unchanged (“null”) genes in relation to the total number of null genes at the chosen statistical cut-off (adjusted p-value). The true positive rate is defined as the number of detected differentially-expressed genes (“non-null”) in relation to the total number of non-null genes at the chosen statistical cut-off. (PDF) [file pone.0030827.s003.pdf]

Figure S3. Statistical analysis of Affymetrix Gene Chips with the LEMMA hierarchical model.

A: Comparison 1 - Electrode vs. Iron(III):

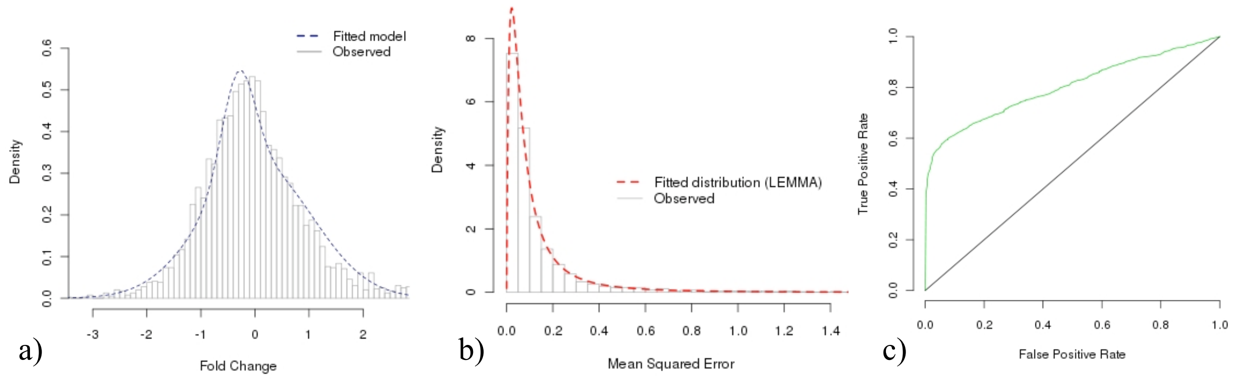

B: Comparison 2 - Electrode vs. Oxygen:

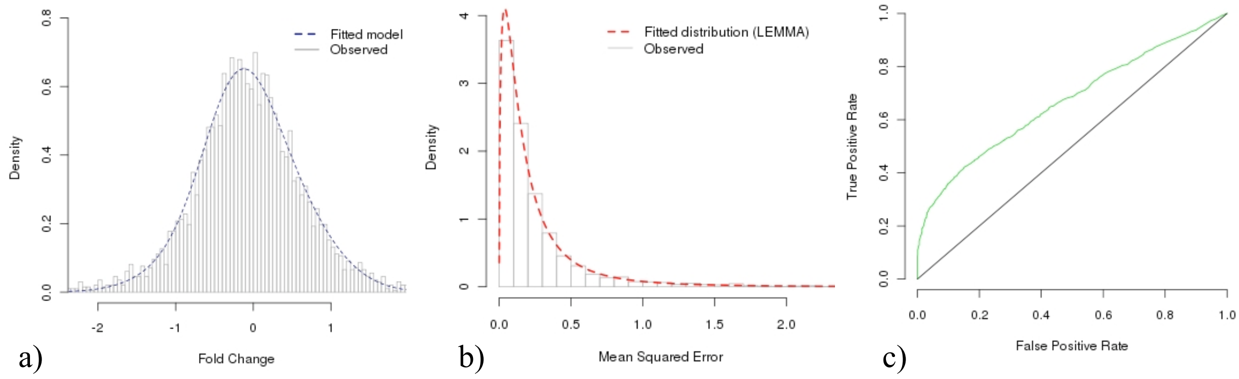

Summary of statistical analysis for comparisons 1(Panel A) and 2 (Panel B): a) Expression effect represented as a Log_2 Fold change (d_g); b) The distribution of gene-specific mean squared error; c) Predicted ratio of false positive to true positive rate for the respective comparison. The false positive rate is defined as the number of detected unchanged (“null”) genes in relation to the total number of null genes at the chosen statistical cut-off (adjusted p-value). The true positive rate is defined as the number of detected differentially-expressed genes (“non-null”) in relation to the total number of non-null genes at the chosen statistical cut-off.
